# Cultural Adaptation and Reliability of the Compliance with Standard Precautions Scale (CSPS) for Nurses in Brazil^1^

**DOI:** 10.1590/1518-8345.1204.2850

**Published:** 2017-03-09

**Authors:** Fernanda Maria Vieira Pereira, Simon Ching Lam, Elucir Gir

**Affiliations:** 2 Adjunct Professor, Universidade Federal Fluminense, Campus Universitário de Rio das Ostras, RJ, Brazil; 3 PhD, Associate Professor, Division of Nursing and Health Studies, The Open University of Hong Kong, Hong Kong, China; 4 PhD, Full Professor, Escola de Enfermagem de Ribeirão Preto, Universidade de São Paulo, WHO Collaborating Centre for Nursing Research Development, Ribeirão Preto, SP, Brazil

**Keywords:** Universal Precautions, Nurses, Validation Studies, Reproducibility of Results

## Abstract

**Objective:**

this study aimed to carry of the cultural adaptation and to evaluate the reliability of the Compliance with Standard Precautions Scale (CSPS) for nurses in Brazil.

**Method:**

the adaptation process entailed translation, consensus among judges, back-translation, semantic validation and pretest. The reliability was evaluated by internal consistency (Cronbach alpha) and stability (test-retest). The instrument was administered to a sample group of 300 nurses who worked in a large hospital located in the city of São Paulo/SP, Brazil.

**Results:**

through the semantic validation, the items from the scale were considered understandable and deemed important for the nurse´s clinical practice. The CSPS Brazilian Portuguese version (CSPS-PB) revealed excellent interpretability. The Cronbach`s alpha was 0.61 and the intraclass correlation coefficient was 0.85.

**Conclusion:**

the initial study showed that CSPS-PB is appropriate to assess compliance with standard precautions among nurses in Brazil. The reliability was considered acceptable. Furhter study is necessary to evaluate its comprehensive psychometric properties.

## Introduction

Standard precautions (SP) are measures for healthcare professionals to minimize the risk of occupational exposure and to ensure patient safety^1^ ^-^ ^3^. Therefore, these measures should be aligned with the task in hand while considering potential exposure to blood and organic fluids^2^. Direct contact with patients, combined with the type and frequency of nursing procedures performed, increase occupational exposure risk of nursing staff to infectious material^4^. However, research studies demonstrated that the use of personal protective equipment (PPE) was still limited among health professionals, especially among nursing staff^5^ ^-^ ^8^. Inappropriate use of gloves indicates poor adherence to hand hygiene^8^ and may increase cross infection^9^. Although hand hygiene is an essential component of SP, studies reveal that health professionals minimally adhere to this practice^8^ ^-^ ^12^. Consequently, it is important to evaluate the compliance of professionals with SP in their clinical practice. However, evaluation is difficult because instruments for comprehensively measuring compliance with SP are mostly in English and have not been translated to or validated to Brazilian Portuguese. In order to study compliance with SP, some authors in Brazil^11^ ^,^ ^13^ and other countries^14^ ^-^ ^15^ have worked on the construction and validation of instruments designed to measure whether professionals are adhering to preventive measures during patient care.

In 2002, a 15-item universal precautions scale was constructed for the purpose of studying the practice of nurses in relation to these measures in Hong Kong^14^. On the basis of this scale, the Compliance with Standard Precautions Scale (CSPS) in English was developed in Hong Kong in 2011 by modifying most items and adding several new ones^15^. This 20-item scale provides a means to assess the compliance of nursing professionals with infection control practices. The CSPS addresses issues related to daily clinical practice, such as the use of protective equipment, disposal of sharps, handling of articles, and prevention of cross infection. After its development, CSPS went through a comprehensive psychometric testing in a group of 453 participants, including nursing staff and students. Results showed that CSPS had satisfactory reliability (i.e., internal consistency and stability), construct validity (i.e., satisfactory results of known-group method and hypothesis testing) and concurrent validity^16^. More importantly, CSPS was subject to a cross-cultural pilot test involving 19 experts from 16 countries^16^. It preliminarily indicated that CSPS is relevant and applicable to most developed and developing regions^16^. Indeed, up to our knowledge, CSPS has been or will be translated to several languages, including but not limited to Arabic, Korean, Mainland, Chinese, Italian, Spanish and Turkish^17^ ^-^ ^18^.

The process of adapting an existing measure has been widely used in different fields because it provides certain benefits, such as saving time and funds, and allowing direct comparisons, which facilitate multicenter research collaboration^19^. Adaptation of CSPS for Brazil is imperative to check the compliance of nurses with SP and facilitate cross-cultural comparisons further on. Several reasons indicate that CSPS is potentially appropriate to be adapted to Brazil. First, CSPS is the only instrument for which cross-cultural pilot testing has been conducted in 16 countries^16^. Such result increases the evidence that the CSPS items will be relevant to Brazil. Second, CSPS is one of instruments that has been adapted to other countries^17^ ^-^ ^18^. Hence, adaption of CSPS would facilitate cross-cultural comparisons and contribute significantly to this research field. Third, the CSPS is developed based on the SP guideline published by WHO and CDC^15^, which is in line with the Brazilian guideline^20^. The CSPS includes indispensable aspects related to compliance with SP regarding the use of protective equipment, recap of needles, prevention of cross-infection from person to person, decontamination of used articles and disposal of waste and sharps. These are also important aspects mentioned in the Brazilian governmental regulatory standard of safety and health^20^. Unlike many instruments that only briefly described the development process, the fourth is that the developer of CSPS provides a detailed instrument development process, a clear explanation of the compliance concept, explicit justification on each developed item^15^, which allows the other researchers to adapt the CSPS to their own cultures. Last, the psychometric properties of CSPS have been comprehensively examined with satisfactory results^16^, which suggest that CSPS is reliable and valid. Thus, it is justified to carry out the cultural adaptation of CSPS and to evaluate its reliability for nurses in Brazil.

## Method

This study consisted of two phases: translation/ adaptation and reliability assessment of the CSPS. Adaptation included the following stages: translation, consensus among judges, back-translation, semantic validation, and pretest^21^. Reliability included analysis of the reliability (internal consistency) and stability (test-retest). The participants were nurses who worked in a large hospital located in the city of São Paulo/SP.

*Translation:* During this stage, two different sworn public translators (i.e., certified public translators) translated the scale from its original language to Portuguese.

*Consensus among judges:* A consensus meeting was held by a committee composed of seven judges, as follows: three nurses specialized in infection control; two certified translators with comprehensive knowledge of English; one researcher in that method; and one researcher from the present study. After the signing of the Free and Informed Consent Form, semantic equivalence, cultural equivalence, and idiomatic equivalence were assessed, and a consensus version of the instrument was formulated.

*Back translation:* The consensus version obtained in the previous stage was translated to English by two independent translators, Americans living in Brazil, in order to compare the quality between the original and translated versions of the scale.

*Semantic validation:* The instruments used for the semantic validation were the following: (1) a form with items including demographic and professional information (gender, date of birth, length of professional experience, sector and work shift, how the person became aware of the SP, infection control courses attended at the hospital); (2) CSPS Brazilian Portuguese version (CSPS-PB) (3) a form to evaluate scale items (Were the questions understandable? Were the questions relevant? What did you understand from these questions? Are the response options in accordance with the questions?). Data were collected by one of the researchers at the workplace at a specific time, and the interview was held after signing of the Free and Informed Consent Form. The duration of the interview was approximately 30 minutes. Participants were randomly selected from a list obtained at the Human Resources Department of the hospital.

*Pretest:* After the cultural adaptation, a pre-test was carried out. The translated and adapted instrument was applied to hospital nurses. It is considered that 30 to 40 is the ideal number for this test^(21)^. The CSPS-PB was applied to 50 nurses who worked at a hospital.

*Field research - Reliability:* The CSPS-PB was applied to 300 nurses working at a large Brazilian public hospital. The inclusion criteria were: being a nurse, acting directly in patient care. The exclusion criteria were: nurse who performed exclusively administrative functions. These criteria were strictly according to the definition of clinical nurse^15^ ^-^ ^16^. Reliability was evaluated by internal consistency and stability. Internal consistency was checked by Cronbach's alpha coefficient (α), ranging from 0 to 1, appropriate coefficients being superior to 0.60 for preliminary investigation^22^ ^-^ ^23^, while coefficients of 0.9 to 0.95 are considered excellent^24^. For the stability, test-retest was used by means of the Intra-class correlation coefficient (ICC). This method is used to verify the correlation between the scores resulting from the first application of the instrument and the second application to the same participants two weeks later. The values used as a reference for such analysis are described as ICC <0.40, indicating a weak correlation; 0.41 <ICC <0.60, moderate correlation; 0.61 <ICC <0.80, good or substantial correlation, and ICC> 0.81 almost perfect or very good^25^ ^-^ ^26^.

*^_Statistical Analysis: IBM(r) SPSS_^* version 19.0 was used for statistical analysis. The significance level was set at p < 0.05 in all analyses.

*Ethical Aspects:* The research proposal received approval from the Research Ethics Committee of the Brazilian institution (CAAE: 13906813.6.3001.5463; Approval number: 599.965-0). The reproduction of CSPS was approved by the developer (Ref: B500D36-201206). Confidentiality and anonymity of the participants were ensured, in accordance with the recommendations of National Health Council Resolution 466/2012. Data were collected voluntarily, as signified by the Free and Informed Consent Form. 

## Results

The adaptation process of the CSPS involved the following stages: translation, consensus among judges, back-translation, semantic validation and pretest.

**Figure 1:**
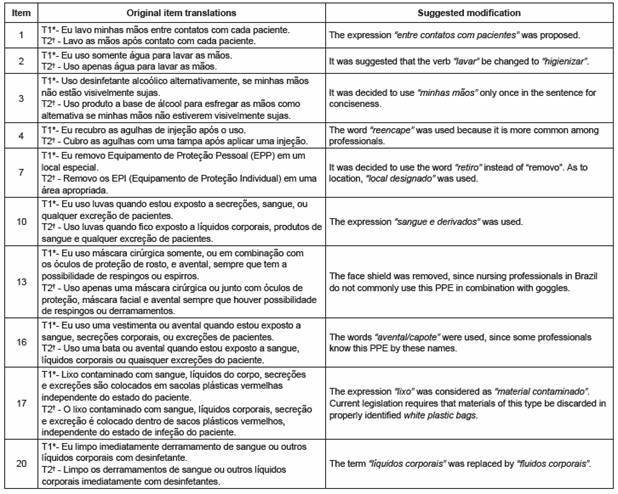
Modifications proposed by the judges to items from the CSPS Brazilian Portuguese version (CSPS-PB). São Paulo, Brazil, 2014

Committee of judges: The committee suggested modifying ten items in the instrument. Their suggestions were accepted when there was 80% agreement among the judges (Figure 1).

The presentation of some items raised questions. In question 16, the word "gown" was added because "apron" may be confused for a white coat when the professional answers this question. Question 20 was extensively discussed in relation to the importance of determining the meaning of "surfaces" and what type of "disinfectant" will be used. However, changes were not made in this item.

*-Back-translation:* A consensus was obtained on the Portuguese version of the scale compared with the original version.

*-Semantic validation:* This stage was performed to check whether all questions were understandable to nurses in the study population. Nurses from a large hospital in the city of São Paulo/SP, Brazil were interviewed. Of these nurses, ten worked in patient care, one was from the Hospital Infection Control Committee, and the other from Continuing Education. All participants evaluated the 20 items in the scale.

The scale was administered to these 12 participants, ten of whom (83.3%) were women. The mean age was 37.3 years (SD = 8.7), with a minimum of 26 and a maximum of 61. With regard to the length of professional experience, six (50%) had been working for ten years or less in nursing. In the overall evaluation, seven (60%) gave a very satisfactory rating of the instrument, whereas five (41.7%) gave a satisfactory rating. None of the participants chose the poor rating. With respect to comprehensibility, ten (83.3%) said that the questions were easy to understand and two (16.7%) answered that they were difficult to understand at times. Insofar as the response options (always, sometimes, seldom, never), 11 (97%) had no difficulty using them and one (8.3%) reported some difficulty. In terms of the relevance of the questions in their daily work, 11 (91.7%) found them to be very relevant and one (8.3%) sometimes relevant.

Altogether, the nurses' evaluation of the scale items revealed that the questions were relevant for their clinical practice and understandable. Furthermore, they expressed that the response options were clear and easy to understand.

Suggested changes to four questions were applied as follows: In question 3 "*Uso produto a base de álcool para esfregar as mãos como alternativa se minhas mãos não estiverem visivelmente sujas* ", the term "*esfregar* " was replaced by "*desinfetar* ." For item 13 "*Uso apenas uma máscara cirúrgica ou junto com óculos de proteção, máscara facial e avental sempre que houver possibilidade de respingos ou derramamentos* ", the word "*apenas* " was removed. For question 17 "*O lixo contaminado com sangue, líquidos corporais, secreção e excreção é colocado dentro de sacos plásticos vermelhos, independente do estado de infeção do paciente* ", the phrase "*Eu descarto* " was added at the beginning of the sentence.

Question 20 raised concerns. The first was related to the type of disinfectant used and the second to the cleaning of surfaces; it was pointed out that nurses are not always responsible for this task. The suggestion made was to cite the type of surface to be decontaminated and the type of product used. Therefore, after consulting with the original author of CSPS about question 20, "*Limpo os derramamentos de sangue ou outros líquidos corporais imediatamente com desinfetantes* ", "(*álcool* )" was added to specify the type of disinfectant used.

*-Pretest*: In this stage, 50 nurses working in patient care at the institution answered the instruments, most of them being women, 40 (80.0%). Regarding the number of jobs, 37 (74.0%) reported having one job and 29 (58.0%) indicated working 30 hours per week. Among the sectors, most of the professionals worked at medical units 34 (68.0%) and 16 (32.0%) at surgical units. All scale items were answered. The CSPS-PB version proved to be understandable and easy to complete. The nurses who participated in this phase did not suggest modifications.

**Figure 2:**
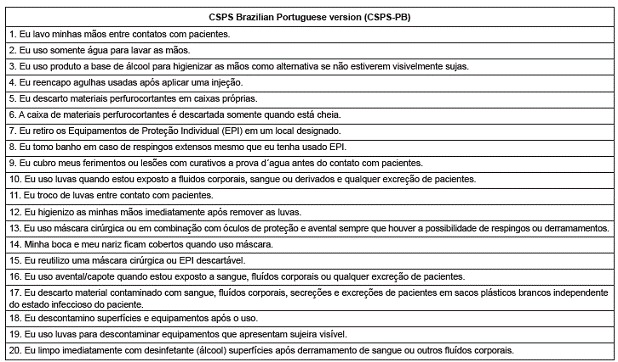
CSPS Brazilian Portuguese version (CSPS-PB). São Paulo, Brazil, 2014

Taking into account all changes that were suggested and made during the stages described above, the CSPS-PB was obtained as presented in Figure 2.

Reliability: The CSPS-PB was applied to 300 nurses working in nursing care at the institution, mostly women (Table 1). The minimum age was 25 years and maximum 75 years.

**Table 1:** Professional and demographic distribution of the nurses (n=300) in the reliability and stability assessment of the CSPS Brazilian Portuguese version (CSPS-PB). São Paulo, Brazil, 2014

Variables		Statistics
Age		χ **^2^** = 39.0 (SD=9.71)
Gender		
	Female	42 (14.0%)
	Male	258 (86.0%)
Standard Precautions Courses		
	No	106 (35.3%)
	Yes	194 (64.7%)
Clinical experience (years)		χ**^2^** = 10,0 (SD=8.60)
Hours worked per week		χ**^2^** = 40,7 (SD=14.81)
Work sector		
	Medical Units	92 (30.7%)
	Surgical Units	77 (25.7%)
	Ambulatory Units	76 (25.3%)
	Intensive Therapy Units	32 (10.3%)
	Emergency Units	23 (7.7%)

The reliability was evaluated by internal consistency (Cronbach alpha) and stability (test-retest). The internal consistency obtained was α=0.61. The ICC obtained for the test-retest with the same participants two weeks later was 0.85 p<0.001, indicating a very good or nearly perfect correlation.

## Discussion

In the process of adapting the CSPS for use in Brazil, most of the nurses considered the instrument very useful and relevant for their daily practice in clinical settings. As for the comprehensibility of the questions, most answered that they were easy to understand.

There were suggestions to modify some questions, particularly item 20, which generated more discussion. Cleaning spills is not a nursing competence in Brazil, but it is an activity executed by the cleaning team. Thus, the review of item 20 was necessary. Another aspect was related to the product used. Although alcohol is considered a disinfectant, this name can also be associated with other products in Brazil (e.g. washing up liquid, dishwashing liquid). In order to reduce confusion and enhance clarity, the word alcohol was added, enclosed in parentheses.

The scope of CSPS includes the use of PPE, waste and sharp disposal, handling of articles and prevention of cross infection. These measures are an immediate concern because current compliance often falls short of recommendations.

Different studies around the world have evaluated the compliance with SP among nursing professionals^6^ ^-^ ^8^ ^,^ ^11^ ^-^ ^16^ ^,^ ^27^. A review of literature for the purpose of identifying instruments already used to assess awareness of and compliance with SP revealed that most of the publications were international; 12 instruments were selected, of which eight have been published in English, three in Portuguese, and one in Spanish^28^. Notably, 66.6% of the studies involved nurses and described the development, origin and construction of the questionnaires. Among the instruments, 58.3% were validated. In another review, 18 instruments were found for verifying compliance with infection control practices. Most of the studies stated that content validity and reliability analyses were performed using internal consistency analysis and test-retest^29^.

The internal consistency was 0.61. Values above 0.60 are considered acceptable for initial validation studies and for research^22^ ^-^ ^23^. The ICC obtained (r=0.85; p<0.001) indicated very good stability^25^ ^-^ ^26^. The previous study of the original version of the CSPS had satisfactory reliability results (Cronbach α = 0.73; ICC = 0.79 for two-week test-retest and 0.74 for three-month test-retest)^16^.

The quality of the adaptation process determines the validity of the instrument to measure the construct in question. Therefore, it is important that an instrument chosen for performing cultural adaption has been well developed and comprehensively validated with satisfactory psychometric properties. Considering the growing number of instrument translation, adaptation, and validation studies performed by nurses, it is necessary to adopt appropriate techniques and methods, and to evaluate reliability and validity, in order to ensure the quality and methodological rigor of the research^30^. The adaptation process used in this study was developed in accordance with the methodological criteria recommended in the literature. It is important to consider the comment given by the developer of a given instrument at every stage and discuss the conceptual meaning of each item in the adaptation process. The developer of CSPS participated in the referred process and approved all changes that were made in the Portuguese version.

## Limitation

Some limitations of this study are noted. The sample for conducting psychometric testing is limited to professional nurses (i.e., nursing staff) at a single hospital institution, which decreases the generalizability of the results. Further study will be valuable if the psychometric properties of CSPS-BP are tested in nursing students or staff working in different types of hospitals. Furthermore, the validity of the CSPS-BP should be reported elsewhere.

## Conclusion

 The CSPS adaptation consisted of the translation, consensus among judges, back-translation, and semantic validation stages. Execution of these stages enabled the cultural adaptation of the CSPS for Brazilian nurses. The CSPS-PB revealed excellent interpretability and items were considered important and relevant to nurses' clinical practices. The reliability was satisfactory. Though the internal consistency value not was considered high, it is acceptable for initial validation studies. The stability was already very good. The initial study showed that CSPS-PB is appropriate to assess compliance with standard precautions among nurses in Brazil. Additional study is needed to evaluate psychometric properties.
